# A naturalistic study on physioterapy in acute psychiatric service

**DOI:** 10.1192/j.eurpsy.2025.1581

**Published:** 2025-08-26

**Authors:** A. Di Luca, A. Tomassini, C. Capanna, S. Tozzi, P. Marignetti

**Affiliations:** 1 Mental Health; 2 Medical, ASL Rieti, Rieti, Italy

## Abstract

**Introduction:**

There is clear evidence on the physical and psychological benefits of a bodily approach for the treatment of psychiatric disorders. They can have a significant impact on the patient’s perceived suffering (Carek et al. IJPM 2011; 41(1) 15–28). In January 2024, a Physiotherapy Project started at the Acute Psychiatric Service of the Rieti ASL in collaboration with the Physical and Rehabilitation Medicine service.

**Objectives:**

Evaluate the impact of the physiotherapy program on psychopathological dimensions and on the patients subjective well-being.

**Methods:**

The naturalistic study was conducted on subjects consecutively hospitalized at the SPDC from January to June 2024 who voluntarily joined the physiotherapy activity. The intervention was administered with bi-weekly sessions of about 45 minutes and included: stretching, orientation exercises, active movement, coordination, muscle strengthening. The Exclusion Criteria were: sedation status, disorganization, behavioral problems. The General Health Questionnaire-12 (GHQ-12) and the Brief Psychiatric Rating Scale (BPRS) were adminstred at admission (T0) and discharge (T1). A Self-evaluation of the useful of the program was administred only to discarege (T1): participants answered by choosing between “not useful”, “partly useful”, “very useful” to 4 questions on the usefullness of the intervention.

**Results:**

Thirty-five participants (17 M, 18 F; mean age 38.2±15,4) were admitted to physical activity. They recieved the following diagnoses: 48.6% Psychotic Disorder, 20% Depressive Disorder, 2.9% Bipolar Disorder, 28.6% Personality Disorder. Eleven of 35 participants had comorbid substance use disorder (14.3% alcohol, 5.7% cocaine, 5.7% cannabis, 2.9% opioids, 2.9% other substances). The hospitalization time was 11.8±4.3 and the average number of physical sessions was 1.7±0.8. The BPRS (44.4±11vs25.9±4.5; F=1024.25; p<0.001) and GHQ-12 (24.6±4.9vs15.11±5.8; F=833,43; p<0.001) mean scores significant improved in two time of evaluation (T0 and T1).

**Table:**
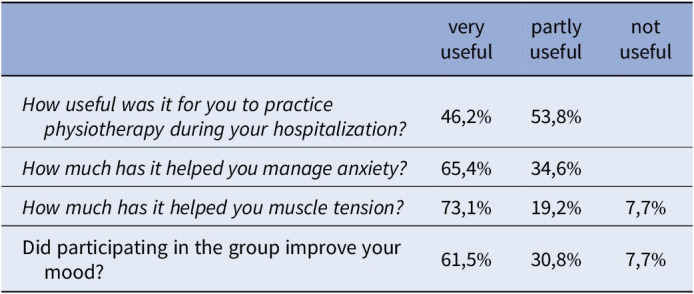

**Conclusions:**

Participants showed a reduction in psychopathological severity and an increase in perceived well-being between T0 and T1. They found useful to practice physioterapy during hospitalozation and to manage anxiety, muscle relaxation and mood improvement.

**Disclosure of Interest:**

None Declared

